# Isolation and Genomic Characterization of Two Lytic *Cutibacterium acnes* Phages Defines Two Novel *Pahexavirus* Species

**DOI:** 10.3390/v18020214

**Published:** 2026-02-06

**Authors:** Anastasia A. Vorobeva, Aleksei M. Vorobev, Peter V. Evseev, Emil R. Mekhtiev, Andrei V. Chaplin, Maria A. Pasivkina, Ekaterina S. Zubkova, Svetlana S. Bochkareva, Mikhail A. Yaitsky, Nikolai A. Nikitin, Natalia B. Demina, Victoria A. Korol, Andrei V. Aleshkin, Anatolij N. Blintsov, Maria N. Anurova

**Affiliations:** 1 G.N. Gabrichevsky Moscow Research Institute for Epidemiology and Microbiology, 125212 Moscow, Russia; vorobjew.alex2010@yandex.ru (A.M.V.); mehtievemil@yandex.ru (E.R.M.); pavlova.maashka@gmail.com (M.A.P.); zubkova@gabrich.ru (E.S.Z.); cip1989@gmail.com (S.S.B.); anurova_m_n@staff.sechenov.ru (M.N.A.); 2 Laboratory of Molecular Microbiology, Pirogov Russian National Research Medical University, 117997 Moscow, Russia; petevseev@gmail.com (P.V.E.); okolomedik@gmail.com (A.V.C.); yaitskiyma@gmail.com (M.A.Y.); 3 Department of Virology, Faculty of Biology, Lomonosov Moscow State University, 119991 Moscow, Russia; nikitin@mail.bio.msu.ru; 4 A.P. Nelyubin Institute of Pharmacy, Sechenov First Moscow State Medical University (Sechenov University), 119991 Moscow, Russia; demina_n_b@staff.sechenov.ru (N.B.D.); dezhurko-korol_v_a@staff.sechenov.ru (V.A.K.); 5 Limited Liability Company Orphan-BIO, 354340 Sirius, Russia; andreialeshkin@googlemail.com; 6 Limited Liability Company Natura Siberica, 119571 Moscow, Russia; a.blintsov@naturasiberica.ru

**Keywords:** *Cutibacterium acnes*, bacteriophages, *Pahexaviruses*, acne, phage therapy, genomic characterization, phenotypic characterization

## Abstract

Bacteriophages are promising antibacterial agents for managing acne vulgaris caused by *Cutibacterium acnes*, particularly given increasing antibiotic resistance. Here, we isolated and characterized two lytic *Cutibacterium* phages, NS-ph1 and NS-ph2, from acne lesions. Both bacteriophages exhibited a broad lytic spectrum, with a high activity against 27 *C. acnes* strains. Adsorption assays indicated rapid attachment and one-step growth experiments revealed latent periods of 4 h (NS-ph1) and 2 h (NS-ph2) and burst sizes of 70 and 59 PFU per infected cell, respectively. After long-term storage at room temperature, both phages retained infectivity for 3 months. Genome sequencing revealed linear dsDNA genomes of 29,490 bp (NS-ph1) and 29,189 bp (NS-ph2) with 51 and 46 predicted ORFs, respectively, and no tRNAs. No genes associated with lysogeny, toxins, or antibiotic resistance were detected. Comparative genomics placed both phages within the genus *Pahexavirus*. Together, these data expand the diversity of *Pahexavirus* and provide two well-characterized lytic candidates for further evaluation in anti-acne phage therapy.

## 1. Introduction

*Cutibacterium acnes* is a lipophilic facultative anaerobe and a commensal opportunistic bacterium in the human skin microbiome [1,2,3]. This Gram-positive, slow-growing bacterium is typical of the coryneform group, with a curved bacillus morphology. The cells measure up to 5 µm in length and approximately 0.7 µm in width [1]. *C. acnes* predominantly colonizes hair follicles and sebaceous glands, and it represents one of the main bacterial etiological factors in the development of acne vulgaris [4].

Acne is a common chronic recurrent inflammatory skin disorder, with a worldwide prevalence of up to 9% [5], and often requires long-term treatment with individualized therapeutic approaches [6,7]. The problem is particularly significant in adolescents, among whom the prevalence can reach 80% [8]. Acne is a multifactorial disease and remains a clinically urgent problem, as it causes not only physical harm but also substantially impairs patients’ quality of life and psycho-emotional wellbeing [6]. The human skin microbiota plays a significant role in acne pathogenesis, with over-colonization by *C. acnes* being a key factor [7,9,10]. For instance, the frequency of *C. acnes* isolation in acne patients can reach 87%, as demonstrated in a cross-sectional study investigating the antibiotic sensitivity of clinical acne isolates [11]. Excessive proliferation of *C. acnes* leads to a degradation of the skin’s microbial diversity. This bacterial overgrowth activates toll-like receptors, provoking the production of pro-inflammatory cytokines (e.g., IL-1α, IL-8, IL-12, TNF-α), antibodies, and enzymes such as lipase, protease, and hyaluronidase. Consequently, the amount of free fatty acids increases and keratinization intensifies. Within the pilosebaceous unit, sebum hypersecretion facilitates this bacterial proliferation, creating a cycle of infection and inflammation that exacerbates disease severity [5,11,12,13].

Beyond acne, the Gram-positive, facultative anaerobic bacillus *C. acnes* has been associated with a range of other infections and diseases. It has been implicated in various device-related infections by colonizing orthopedic implants [14,15,16], cardiac implants [17], contact lenses [18], and cerebrospinal fluid drainage tubes. Furthermore, cases have been reported linking this bacterium to progressive macular hypomelanosis [19], endophthalmitis [20], endocarditis [21], and postoperative central nervous system infections [22]. Additionally, *C. acnes* has been linked to or associated with thyroid cancer [23] and prostate cancer [24,25].

The traditional approach to treating infections caused by *C. acnes* involves topical and systemic antibiotic therapy [26]. However, their use is accompanied by adverse effects, disruption of the normal microbiota, and reduced efficacy due to the growing problem of antibiotic resistance [27]. Resistance, including multidrug resistance, is commonly reported against prescribed antibiotics such as erythromycin, clindamycin, doxycycline, tetracycline, trimethoprim/sulfamethoxazole, levofloxacin, minocycline, etc. [11,27,28,29]. Excessive and uncontrolled use of antibacterial drugs promotes the formation of bacterial biofilms as a defense mechanism, which enhances bacterial tolerance to environmental stressors [30]. These biofilms form a complex barrier and influence disease severity [13,31,32]. The ability of acne-associated *C. acnes* strains to form biofilms has been demonstrated in multiple studies [30,32,33,34].

Antibiotic resistance is now recognized as a critical global health issue that demands urgent scientific attention [27,35,36,37]. Therefore, the development of effective and safe alternative therapies is essential to combat antibiotic-resistant *C. acnes*, including its biofilm-associated forms. One promising strategy is bacteriophage therapy [4,13,38]. The advantages of bacteriophages over antibiotics include their activity against antibiotic-resistant strains, preservation of the normal microbiota due to high specificity, compatibility with many medications, and a potential immunomodulatory effect [13]. Numerous in vitro and in vivo studies have demonstrated that phages can effectively reduce *C. acnes* levels [4,39,40,41,42,43].

The aim of this study was to isolate novel bacteriophages with lytic activity against a broad range of *C. acnes* strains and to characterize their phenotypic and genetic properties to develop a safe and effective antibacterial agent for treating *C. acnes*-associated infections.

## 2. Materials and Methods

### 2.1. Bacterial Strains, Culture Conditions, and Identification of Clinical Isolates

A total of 25 *Cutibacterium acnes*, 5 *C. granulosum*, and 7 *C. avidum* clinical isolates obtained from 43 acne patients, as well as *C. acnes* ATCC 6919 and ATCC 11827 reference strains, were used in this study. Skin samples were collected using sterile dry swabs in charcoal transport medium (Yancheng Huida Medical Instruments Co., Jiangsu, China). After sampling, the inoculated swabs were incubated at 37 ± 2 °C for 3 days and subsequently streaked onto Columbia agar (Research Center for Pharmacotherapy (RCP), St. Petersburg, Russia) supplemented with 5% defibrinated mutton blood (RCP, St. Petersburg, Russia). Anaerobic cultivation was performed using either the GasPak EZ system (BD Diagnostics, Allschwil, Switzerland) or the Bactron 300-2 anaerobic workstation (Sheldon Manufacturing Inc.,Cornelius, OR, USA) under a gas mixture containing 10% CO_2_, 10% H_2_, 80% N_2_ at 37 ± 2 °C for 2–5 days.

Clinical isolates were identified by matrix-assisted laser desorption/ionization time-of-flight mass spectrometry (MALDI-ToF MS) using the BactoSCREEN microbiological analyzer (Lytech LLC, Moscow, Russia) according to the manufacturer’s instructions. The master culture collection of *C. acnes* strains was stored in glycerol at −80 ± 2 °C.

### 2.2. Antibiotic Susceptibility Testing

Antibiotic susceptibility of the clinical isolates was assessed using the disk diffusion method. The following antibiotic disks (RCP, St. Petersburg, Russia) were used: azithromycin (15 µg), ampicillin (10 µg), gentamicin (10 µg), doxycycline (30 µg), clarithromycin (15 µg), clindamycin (2 µg), levofloxacin (5 µg), tetracycline (30 µg), erythromycin (15 µg), and ciprofloxacin (5 µg). The inhibition zone diameters were measured after incubation, and the isolates were classified as resistant, intermediate, or susceptible according to the manufacturer’s interpretive criteria.

### 2.3. Bacteriophage Isolation and Culturing Conditions

*Cutibacterium acnes* phage NS-ph1 (hereafter NS-ph1) and *Cutibacterium acnes* phage NS-ph2 (hereafter NS-ph2) were isolated from clinical acne samples using clinical *C. acnes* isolates 12.1 and 2.1 as host strains, respectively, via an enrichment method. A 4.5 mL aliquot of BHI broth (HiMedia Laboratories Pvt. Ltd., Mumbai, India) was added to the swab-containing transport medium. Then, 0.2–0.5 mL of a 24 h culture of the corresponding host strain (*C. acnes* 2.1 or *C. acnes* 12.1) was added. The swabs were incubated anaerobically at 37 ± 2 °C for 48–72 h. Afterwards, the suspensions were filtered through a syringe filter (PES membrane, pore size 0.22 μm). The resulting filtrates were screened for bacteriophages active against the host *C. acnes* strains using the spot-test assay. For this, cultures of *C. acnes* 2.1 and 12.1 were spread in a continuous bacterial lawn on 5% blood agar. Then, 20 µL of filtrate was applied to the surface. Plates were incubated anaerobically for 72 h, and plaques (clear zones of host cell lysis) were observed. The clear lysis areas were excised along with the agar and placed in tubes with PBS. Agar plugs containing individual plaques were excised, placed in PBS, and vortexed for 30 min at 160 rpm (PSU-20i shaker, Biosan, Riga, Latvia). The suspensions were then centrifuged at 5000× *g* for 30 min (centrifuge Megafuge 1.0R, Heraeus, Hanau, Germany). The supernatant was collected, filtered through a 0.22 μm syringe filter, and phages were propagated using the double-agar overlay method (Gratia’s method) on Petri dishes with 1.5% agar containing brain heart infusion (Himedia Laboratories Pvt. Ltd., Mumbai, India). Plates were incubated at 37 ± 2 °C under anaerobic conditions for 48–72 h. To obtain pure bacteriophage isolates, material from a single, well-defined plaque was resuspended into PBS. Ten-fold serial dilutions were prepared for repeated double-agar overlay plaque purification. These manipulations were repeated five times to guarantee clonality of the bacteriophage. Plaque morphology was visually assessed. Sterile phage lysates were stored in PBS at 4 ± 2 °C and in glycerol at −80 ± 2 °C.

### 2.4. Determination of Bacterial Host Range and Efficacy of Plating (EOP)

The host range of each phage was evaluated by a spot-test assay using the panel 27 of *C. acnes* strains. A 20 μL aliquot of phage lysate was applied to each bacterial lawn and incubated under anaerobic conditions at 37 ± 2 °C for 72 h.

Quantitative bacterial susceptibility to the bacteriophages was assessed by determining the efficiency of plating (EOP). Briefly, 100 µL of an overnight culture of each *C. acnes* isolate was mixed with 3.5 mL of warm BHI soft agar (0.7%) and poured onto a base layer of BHI agar (1.5%). After the top agar solidified at room temperature, a 30 µL aliquot of serial dilutions of the phage suspension (from 10^1^ to 10^7^ PFU/mL) was spotted onto each bacterial lawn. Plates were incubated anaerobically at 37 ± 2 °C for 72 h. The EOP was calculated as the ratio of phage titer (PFU/mL) on the tested strain to the titer on the corresponding host strain (*C. acnes* 12.1 for NS-ph1; *C. acnes* 2.1 for NS-ph2). Based on this value, phages were classified as highly active (0.100 < EOP ≥ 1.000), moderately active (0.001 < EOP < 0.099), weakly active (EOP < 0.001), or inactive (no plaques detected).

### 2.5. Determination of the Specificity of Bacteriophages

The specificity of the isolated bacteriophages targeting *C. acnes* was evaluated using a spot-test assay on cultures of related *Cutibacterium* species: *C. granulosum* and *C. avidum*. These strains were clinical isolates obtained from patients with acne.

### 2.6. Electron Microscopy of Bacteriophages

The morphology of phage particles was examined by transmission electron microscopy (TEM) using negative staining. Samples were imaged with a JEM-1011 transmission electron microscope (JEOL, Tokyo, Japan) operated at an accelerating voltage of up to 80 kV. Micrographs were captured using a Gatan Erlangshen ES500W (Gatan, Pleasanton, CA, USA) side-mounted digital camera controlled by Digital Micrograph v.3 software (Gatan, Pleasanton, CA, USA). Phage particle dimensions were measured using ImageJ v.1.54d software (NIH, Bethesda, MD, USA).

### 2.7. DNA Extraction and Genome Sequencing

Phage genomic DNA was extracted using the ExtractDNA Blood&Cells DNA extraction kit (Evrogen, Moscow, Russia) according to the manufacturer’s protocol. DNA concentration was quantified using a Qubit^®^ 2.0 fluorimeter (Invitrogen, Carlsbad, CA, USA). Whole-genome sequencing was performed on an Illumina MiSeq platform (Illumina, San Diego, CA, USA) using 2 × 150 bp paired-end reads. Sequencing libraries were prepared with the SG GM DNA library kit (Raissol Bio, Moscow, Russia). Finally, the reads were assembled into contigs using SPAdes v4.2.0 [44].

### 2.8. Adsorption Analysis and One-Step Growth Curve

For the adsorption assay, exponentially growing bacterial cells were mixed with phage suspension at an MOI of 0.001 and incubated at room temperature. Aliquots of 100 µL were collected every minute from 1 to 20 min, mixed with 850 µL of SM buffer and 50 µL of chloroform, and placed on ice. Samples were vortexed using a Microspin FV-2400 (Biosan, Riga, Latvia) at 2800 rpm and centrifuged at 7000× *g* for 10 min using a BKC-MH20-B centrifuge (Biobase, Jinan, China). The number of non-adsorbed or reversibly adsorbed phage particles in the supernatant was determined by the Gratia method. SM buffer containing the initial phage suspension but lacking host bacteria served as a control. The percentage of non-adsorbed (free) phages was calculated as: [residual titer/control titer] × 100%.

The one-step growth curve was determined based on a previously described protocol [43] with minor modifications. Exponentially growing strains of *C. acnes* 12.1 and *C. acnes* 2.1 (OD600 = 0.5) were infected with NS-ph1 and NS-ph2, respectively, at an MOI of 0.001 in BHI broth and allowed to adsorb at 4 ± 2 °C for 60 min. The mixtures were centrifuged at 12,000× *g* for 5 min, and the bacterial precipitate was resuspended in 10 mL of fresh BHI broth. Cultures were then incubated at 37 ± 2 °C under anaerobic conditions. Samples were collected every 2 h for a total of 12 h and immediately titrated using a double-agar overlay method. All experiments were performed in biological triplicate.

### 2.9. Stability Tests

To assess temperature stability, phage preparations were exposed to temperatures of −80 ± 2 °C, −20 ± 2 °C, 4 ± 2 °C, 25 ± 2 °C, 37 ± 2 °C, and 45 ± 2 °C for up to 3 months. Phage titers were measured immediately after mixing, and after 1 day, 3 days, 1 week, 1 month, and 3 months. For the pH stability assay, the following buffer solutions were used: citrate buffer (pH 4.5 and 5.5) and phosphate-buffered saline (PBS) (pH 6.5 and 7.5). Phage titers were determined immediately after mixing and after 1 day, 3 days, 1 week, 1 month, and 3 months of incubation. Following exposure to the different pH conditions, phage viability was assessed by plaque formation using the Gratia method. To assess chloroform stability, the phage lysates were mixed with chloroform at a 1:10 ratio and shaken at 170 rpm for 45 min at room temperature. For the control, PBS buffer was added instead of chloroform. The mixtures were then centrifuged to separate the aqueous phase from the chloroform layer, and the phage titers were subsequently determined. The stability of phage NS-ph1 was studied using *C. acnes* 12.1 as the host strain, while the stability of phage NS-ph2 was studied using *C. acnes* 2.1 as the host strain.

### 2.10. Statistical Analysis

All experiments were performed in biological triplicate, and the results were expressed as an average value ± standard deviation. Descriptive and statistical data analysis was performed and visualized using GraphPad Prism 10.4.2.

### 2.11. Bioinformatic Analyses

Open reading frames (ORFs) in phage genomes were identified using Glimmer v3.02b [45] and Prodigal v2.6.3 [46]. Gene boundaries were manually curated. Functional annotation of the predicted genes was performed using a combination of sequence similarity and structure-based approaches. First, BLAST+ v2.12.0 searches [47] were conducted against the NCBI nr/nt and GenBank PHG databases (https://www.ncbi.nlm.nih.gov, accessed on 10 December 2024). Then, HHpred [48] searches were performed against the PDB70_mmCIF70, PfamA-v37, UniProt-SwissProt-viral70_3, and NCBI_Conserved Domains (CD) databases. Genome termini were analyzed using PhageTerm v1.0.12 [49].

Multiple sequence alignments of nucleotide and protein sequences were produced with Clustal Omega v1.2.3 [50] using the full distance matrix option. Phylogenetic inference from the resulting alignments was carried out in IQ-TREE v2.2.5 [51] with the command-line options “-m TEST -ninit 1000 -bb 1000”. Under this configuration, ModelFinder [52] was used to select the best-fitting substitution model prior to tree reconstruction. Intergenomic similarity comparisons among phages were performed with VIRIDIC v1.1 [53] using the default settings. All phylogenetic trees were visualized and annotated in iTOL v8 [54].

Protein structures were predicted with AlphaFold 3 [55] and rendered in PyMOL v2.5.4 (Schrödinger Inc., New York, NY, USA). Structural analyses and pairwise comparisons were based on the highest-ranked AF3 models. Protein structural similarity was additionally evaluated by DALI using Z-scores [56]. Putative lysogeny-associated proteins were screened using HHblits v3.3.0 [57] with default parameters against the databases pdb70_from_mmcif, pfama-v35, and uniprot_sprot_vir70, followed by a targeted keyword search for “excisionase”, “integrase”, “recombinase”, and “repressor” and subsequent manual verification of candidate hits.

## 3. Results

### 3.1. Bacteriophage Isolation and Host Range

Two bacteriophages were isolated from 43 biomaterial samples obtained from patients with acne using clinical isolates *C. acnes* 12.1 and *C. acnes* 2.1 as host strains. The lytic spectrum of the isolated bacteriophages was assessed against 25 clinical and two reference strains. Both phages, NS-ph1 and NS-ph2, demonstrated lytic activity against all tested *C. acnes* strains under the experimental conditions used in this study (Table 1).

According to the antibiotic resistance testing, 28% of the isolates were resistant to clindamycin, 24% to azithromycin, 16% to clarithromycin, 12% to gentamicin, 8% to erythromycin, and 4% to ampicillin.

### 3.2. Bacteriophages Specificity

The results demonstrated that bacteriophages NS-ph1 and NS-ph2 were strictly specific for *C. acnes* strains. No lytic activity was detected against the closely related species *C. granulosum* and *C. avidum*. These data indicate a high degree of phage selectivity, supporting their potential use as targeted therapeutic agents against *C. acnes* infections without disrupting other components of the skin microbiota.

### 3.3. Morphological Analysis

**Figure 1:**
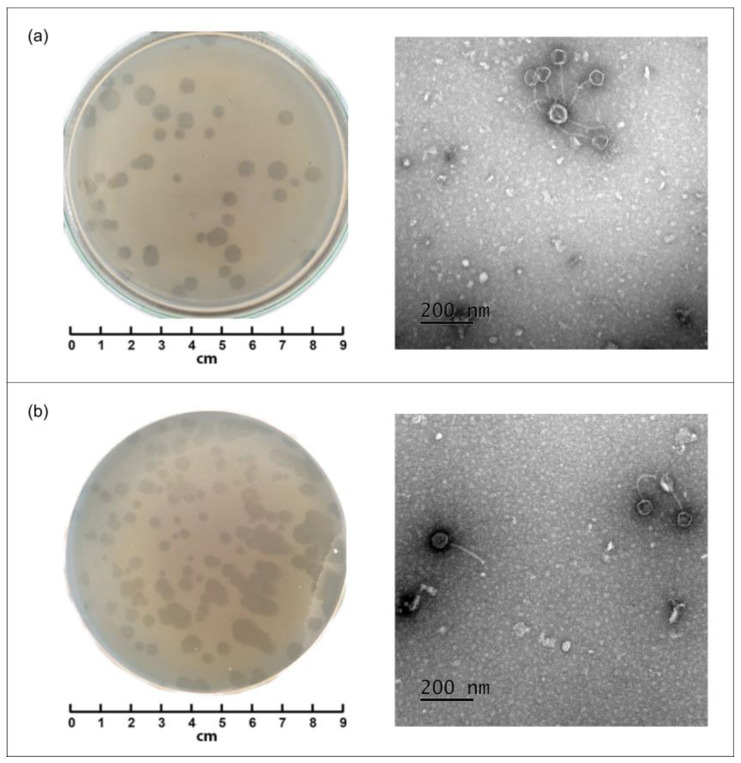
Plaque morphology of the isolated phages and transmission electron microscopy (TEM) images (**a**) plaques and TEM of NS-ph1 phage (host strain *C.acnes* 12.1), (**b**) plaques and TEM of NS-ph2 phage (host strain *C.acnes* 2.1).

Negative colonies of phages NS-ph1 and NS-ph2 were evaluated using their respective host strains, *C. acnes* 12.1 and *C. acnes* 2.1, by the Gratia method. Both phages produced clear, round lytic plaques with diameters ranging from 2 to 7 mm (Figure 1a,b).

TEM of phages revealed that NS-ph1 possesses an isometric head measuring 62 ± 5 nm and a tail length of 166 ± 5 nm (Figure 1a). Phage NS-ph2 exhibited a similar morphology, with a head size of 59 ± 5 nm and a tail length of 160 ± 5 nm (Figure 1b). The phages had long, flexible, non-contractile tails, characteristic of siphovirus-like morphology, and both exhibited similar structural features. Phage aggregates were readily observed under TEM.

### 3.4. Adsorption Rate and One-Step Growth Curve of Phages

**Figure 2:**
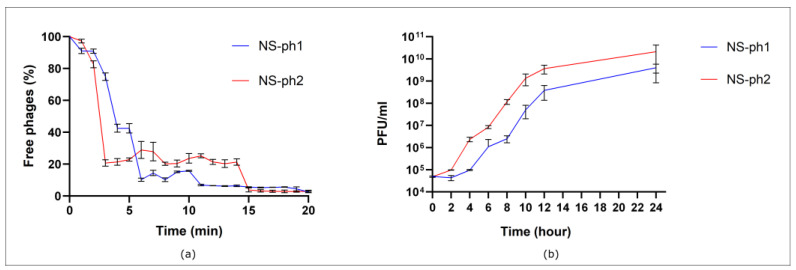
Adsorption assay (**a**) and one-step growth curve (**b**) of phage NS-ph1 using *C. acnes* 12.1 as the host strain, and phage NS-ph2 using *C. acnes* 2.1 as the host strain.

According to the research results, the adsorption of bacteriophages on the cells of target bacteria is significantly extended over time. For NS-ph1, approximately 89% of phage particles adsorbed within the first 6 min, after which the rate decreased, reaching complete adsorption (90–100%) by 13 min. Phage NS-ph2 adsorbed slightly faster, with 80% of particles binding within the first 3 min and full adsorption (90–100%) achieved by 15 min (Figure 2a).

The one-step growth experiments revealed that phage NS-ph1 exhibited a latent period of approximately 4 h in *C. acnes* 12.1, with an average burst size of 70 virions per infected cell. In contrast, phage NS-ph2 displayed a shorter latent period of 2 h in *C. acnes* 2.1 and a burst size of 59 virions per cell.

### 3.5. Phage Stability

Both phages demonstrated complete resistance to chloroform treatment: no significant reduction in titer was observed after 15, 30, or 45 min of exposure, with all fluctuations falling within the methodological error. These results indicate that the phages are chloroform-resistant. Consequently, chloroform can be used for removing non-lysed bacteria during phage propagation and purification.

**Figure 3:**
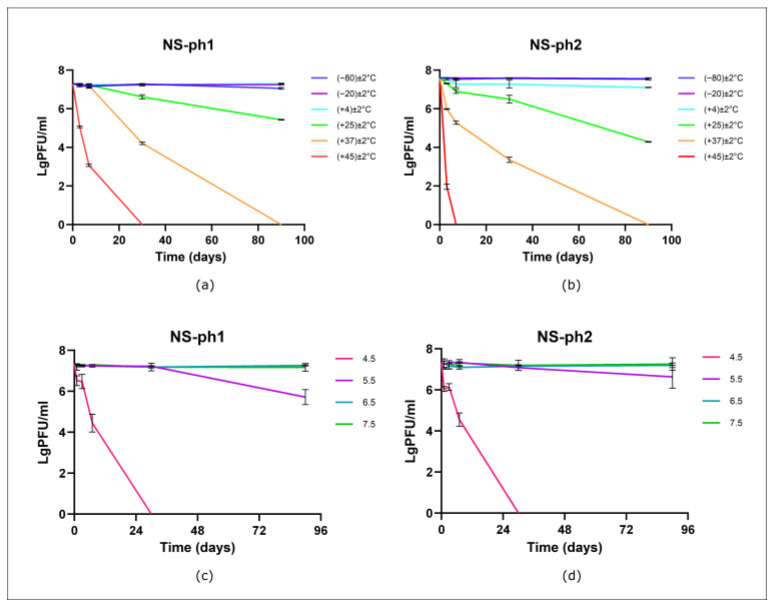
Stability of bacteriophages NS-ph1 and NS-ph2 under (**a**,**b**) different temperatures and (**c**,**d**) different pH. (The experiments were performed for phage NS-ph1 using *C. acnes* 12.1 as the host strain, and for phage NS-ph2 using *C. acnes* 2.1 as the host strain).

Experiments on phage stability during storage indicated that after three months of storage, lytic activity was fully preserved in frozen (−80 ± 2 °C) and (−20 ± 2 °C) and refrigerated (4 ± 2 °C) samples. In contrast, storage of phage lysates at room temperature (25 ± 2 °C) resulted in a reduction in phage titers. At the three-month time point, 74.39% of NS-ph1 and 55.98% of NS-ph2 virions retained lytic activity (Figure 3a,b).

The stability of bacteriophages NS-ph1 and NS-ph2 at different pH values was assessed over a three-month period (Figure 3c,d). Both phages remained stable in the pH range of 5.5–7.5, with 80–100% of virions maintaining lytic activity.

### 3.6. General Genomic Features

Genomes of *Cutibacterium* phages NS-ph1 and NS-ph2 comprise double-stranded DNA molecules of 29,490 and 29,189 base pairs, respectively (NCBI accession numbers PV990964 and PV990965). The GC-content of NS-ph1 is 54.3% and the GC-content of NS-ph2 is 54.1%; this is somewhat lower than that of the bacterial host *C. acnes* (~60.1% for the type strain ATCC6919 [58]). Gene calling predicted 51 ORFs in NS-ph1 and 46 ORFs in NS-ph2, and no tRNA genes were found in the genomes. No lysogeny-associated genes, as well as no genes encoding toxins or other virulence factors, and no antibiotic-resistance determinants were detected. PhageTerm analysis supported fixed genome termini with 3′-cohesive ends (COS (3′), HK97-type) for both phages, i.e., a non-permuted genome configuration with defined physical ends rather than headful packaging.

**Figure 4:**
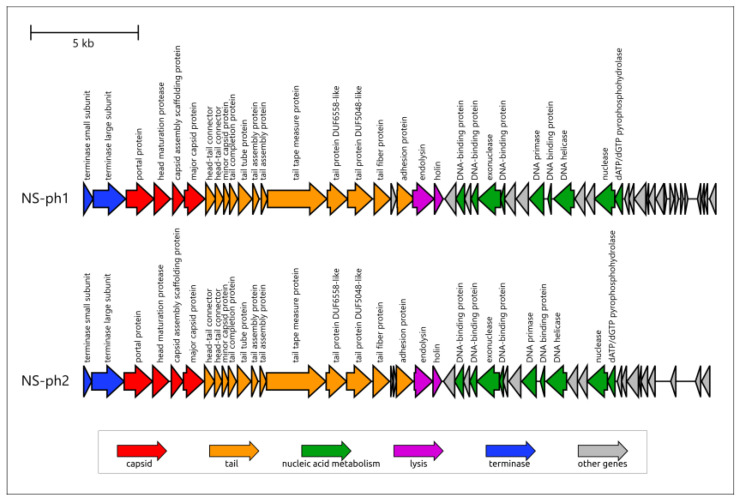
Genome map of *Cutibacterium* phages NS-ph1 and NS-ph2. Coding sequences are colored based on their general functions, arrows’ directions represent the direction of transcription.

The genomes of phages NS-ph1 and NS-ph2 exhibit a distinct modular structure (Figure 4). The packaging module, comprising small and large terminase subunits (TSS and TLS), is followed by the capsid (head) module, the tail module, the lysis block, the replication module, and the block of genes possibly responsible for the early stages of infection. The gene architecture and the list of genes are nearly identical for both phages; the differences were predicted mainly for the putative early genes. The capsid (head) module comprises genes encoding the portal protein, maturation protease, scaffolding protein, and major capsid protein (MCP), typical of HK97 phages exploiting the protease-scaffold-MCP assembly process [59]. No head decoration protein genes were found in the genomes. The composition of the tail module is consistent with the morphology of siphoviruses and includes a tail tape measure protein but does not include tail sheath proteins. The tail module contains relatively few genes compared with large siphoviruses. This module was predicted to include two proteins participating in the adsorption stage, namely the tail fiber protein (TFP) and a putative so-called “adhesion protein”. Overall, accounting for siphovirus morphology, the architecture of the structural block looks simple, as in other small phages, e.g., *Bacillus* phage phi29 [60], featuring a simplified architecture and the absence of duplicated genes. The lysis block was found to include an endolysin and a holin; no spanin genes, characteristic of phages infecting Gram-negative bacteria, were found in the genomes. The replication module is adjacent to the lysis block and is oriented in the direction opposite to the packaging, structural, and lysis modules. The replication module is similar in size to the structural block and is rich in diverse genes, including those encoding DNA primase, helicase, nucleases, and multiple DNA-binding proteins.

### 3.7. Phylogeny and Taxonomy

**Figure 5:**
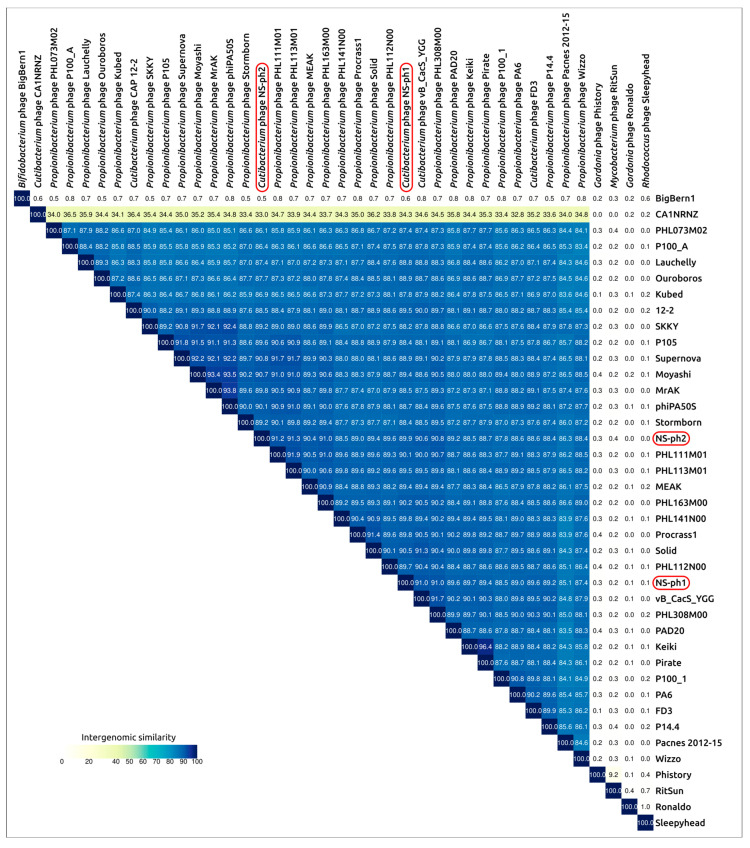
VIRIDIC heatmap generated using genomic sequences of the phages related to *Cutibacterium* phages NS-ph1 and NS-ph2.

BLAST searches using the genomic sequences of phages NS-ph1 and NS-ph2 against the NCBI core nucleotide collection and other GenBank databases, as well as searches using the MCP and TLS sequences, indicated that the closest known phages belong to the genus *Pahexavirus*, which is currently assigned directly to the class *Caudoviricetes* [61]. Calculations of intergenomic similarity using genomic sequences of different pahexaviruses, together with more distantly related unclassified phages *Cutibacterium* phage CA1NRNZ and *Bifidobacterium* phage BigBern1, indicated distinct clustering of phages NS-ph1 and NS-ph2 within the *Pahexavirus* cluster, with similarity values of 85.11–91.04% and 86.09–91.28%, respectively (Figure 5). The closest phage to NS-ph1 is *Cutibacterium* phage vB_CacS_YGG, whereas the closest phage to NS-ph2 is *Cutibacterium* phage PHL113M01 (classified by the ICTV as *Pahexavirus* PHL113M01). The similarity to phage PA6 [62], which gave its name to the genus *Pahexavirus*, is 88.93% and 88.61% for phages NS-ph1 and NS-ph2, respectively. Thus, taking into account the ICTV-recommended cut-offs for bacterial viruses (a 95% species cut-off and a 70% genus cut-off), both phages can be assigned to new species within the genus *Pahexavirus*. Notably, our searches also revealed a more distantly related and currently unclassified *Cutibacterium* phage CA1NRNZ, which cannot be assigned to the genus *Pahexavirus* but has a similar genomic size and architecture.

**Figure 6:**
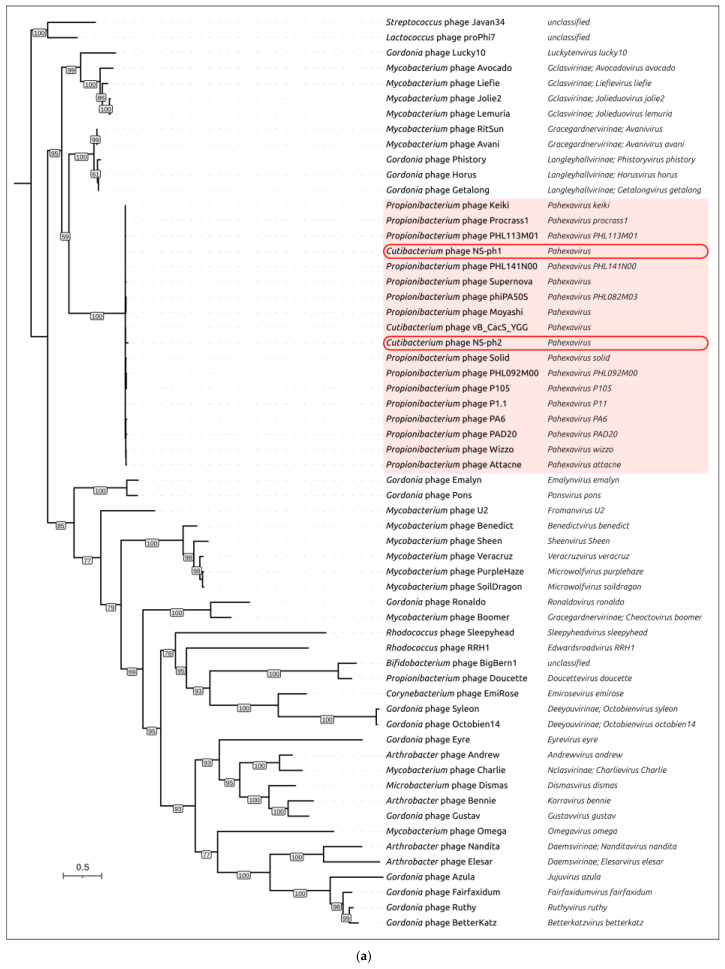

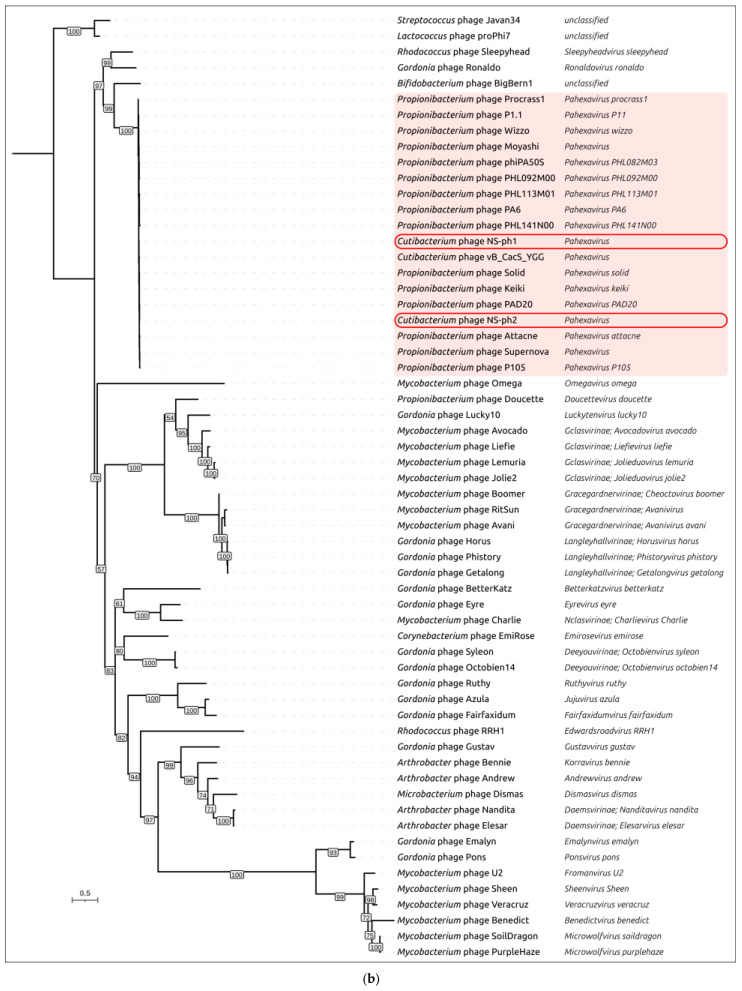
Maximum-likelihood phylogenetic trees inferred from 100 representative amino acid sequences of (**a**) major capsid protein and (**b**) the ATPase domain of the terminase large subunit. Taxonomic assignments are shown in the tip labels and color-shaded blocks in the legend. Phages NS-ph1 and NS-ph2 are highlighted with a red rounded box. *Pahexavirus* clade is colored light pink. Both trees are rooted to *Streptococcus* phage Javan34 and *Lactococcus* phage proPhi7. Bootstrap support values are shown next to the corresponding nodes; nodes with bootstrap support lower than 50% collapsed into polytomies. The scale bar indicates the expected number of substitutions per site.

Phylogenetic analysis was conducted using the sequences of MCP and TLS (analyzed separately for the ATPase and endonuclease domains). Phylogenetic reconstruction based on major capsid protein (MCP) sequences placed *Cutibacterium* phages NS-ph1 and NS-ph2 within a single, coherent clade comprising *Pahexavirus* representatives (Figure 6a), whereas more distant and currently unclassified actinophages were resolved outside this cluster. Within *Pahexavirus*, NS-ph1 and NS-ph2 did not form a sister pair in the MCP tree. Instead, each phage was embedded in a different, but close, sublineage of the genus, basically consistent with their distinct closest relatives inferred from intergenomic similarity. Overall, the MCP phylogeny supports assignment of both phages to *Pahexavirus*. The phylogeny inferred from the ATPase domain of the large terminase subunit (Figure 6b) yielded a congruent genus-level placement: both NS-ph1 and NS-ph2 again grouped within *Pahexavirus*, clearly separated from the more distantly related actinophages. Together with the VIRIDIC-based intergenomic similarity values, these phylogenies provide independent support that both isolates belong to *Pahexavirus*. However, the topologies of the MCP and TLS-ATPase trees are not fully congruent beyond the genus level, indicating a complex evolutionary history of these phages that may include recombination events [63]. Interestingly, the TLS-ATPase tree and the TLS-endonuclease tree (Supplementary Figure S1) also show dissimilarities in their topologies, although these are less pronounced than those observed between the MCP and TLS-ATPase trees.

**Figure 7:**
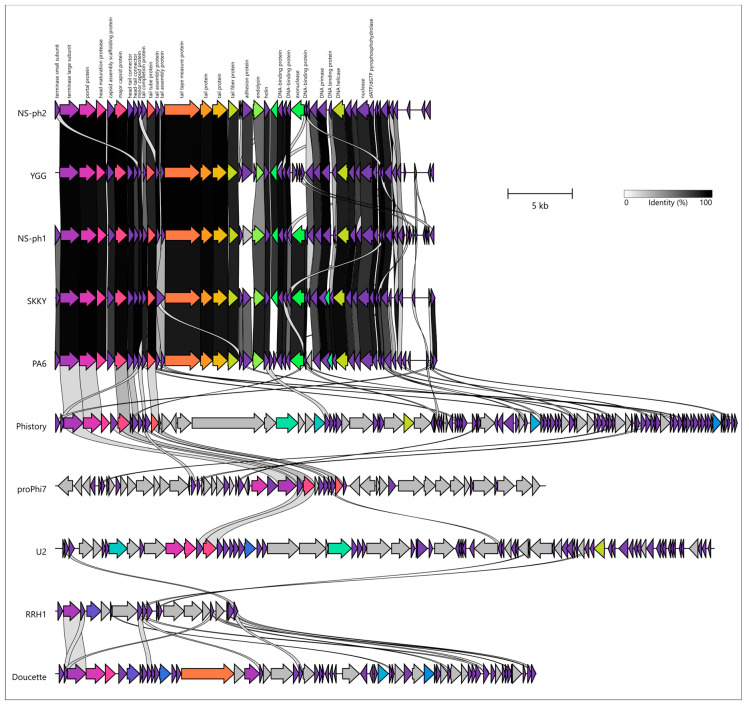
Whole-genome synteny comparison of phages NS-ph1 and NS-ph2 with representative *Pahexavirus* members and more distantly related actinophages. Predicted ORFs are shown as arrows (arrow direction indicates transcriptional orientation). Gray-to-black shading between genomes denotes nucleotide identity (0–100%), and curved connectors indicate homologous genes. Scale bar, 5 kb.

Analysis of whole-genome synteny was conducted using BLAST homology searches between the predicted proteins encoded by phages NS-ph1 and NS-ph2 and those encoded by pahexaviruses and other phages (Figure 7). Whole-genome synteny comparisons revealed that NS-ph1 and NS-ph2 are highly colinear with the other *Pahexavirus* genomes included in the analysis (*Cutibacterium* phage vB_CacS_YGG, SKKY, and the reference *Pahexavirus* PA6). Across this group, the packaging and virion morphogenesis module shows a conserved gene order and extensive regions of high sequence similarity, spanning the canonical terminase small and large subunits, portal protein, head maturation protease, capsid assembly scaffolding protein, major capsid protein, head-tail connector/adaptor proteins, and a continuous tail morphogenesis block including the tail tube, tape measure protein, and other tail proteins. However, the tail fiber protein appears somewhat less conserved than the other structural proteins, and the adhesion protein varies markedly within the analyzed *Pahexavirus* cluster, in agreement with its participation in the first stages of phage-host interactions [64,65]. The lysis cassette (endolysin and holin) is conserved in position and remains linked to the late structural region; however, there are apparent changes in the *Pahexavirus* endolysin sequences, possibly indicating adaptations to hosts. In contrast, the more distantly related actinophages included for comparison (*Gordonia* phage Phistory belonging to the family Langleyhallvirinae, unclassified *Lactococcus* phage proPhi7, *Mycobacterium* phage U2 of the genus *Fromanvirus*, unclassified *Rhodococcus* phage RRH1, and *Propionibacterium* phage Doucette of the genus *Doucettevirus*) displayed only limited, patchy similarity, mainly restricted to parts of the packaging and structural module (TLS and capsid genes), whereas the remainder of their genomes differed strongly in gene content and organization. This pattern highlights a pronounced synteny break between *Pahexavirus* genomes and the more distant reference phages, together with the modular evolution accompanying bacteriophage diversification [66,67].

### 3.8. Structural Analysis of Phages Proteins

**Figure 8:**
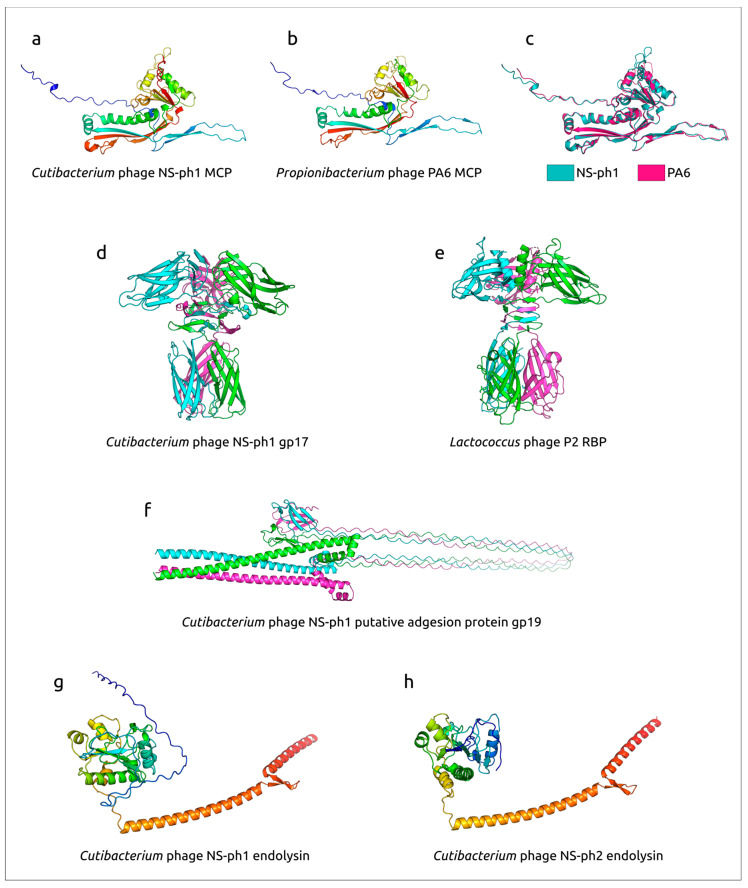
Structural modeling of selected proteins of *Cutibacterium* phages NS-ph1 and NS-ph2. (**a**) AlphaFold-predicted structure of the major capsid protein (MCP) of *Cutibacterium* phage NS-ph1. (**b**) Experimentally determined MCP of *Propionibacterium* phage PA6 (PDB: 3JB5, chain A). (**c**) Structural superposition of the NS-ph1 MCP model (cyan) and the PA6 MCP structure (magenta). (**d**) AlphaFold model of the putative tail fiber protein gp17 of phage NS-ph1 (modeled as a homotrimer). (**e**) Experimentally determined trimeric receptor-binding protein (RBP) of *Lactococcus* phage P2. (**f**) AlphaFold model of the putative adhesion protein gp19 of phage NS-ph1 (modelled as a homotrimer). (**g**,**h**) AlphaFold-predicted structures of the endolysins of phages NS-ph1 (**g**) and NS-ph2 (**h**). Monomeric models (**a**,**b**,**g**,**h**) are colored by sequence position (blue, N-terminus; red, C-terminus). Trimeric models (**d**,**f**) are shown with each subunit in a different color.

AlphaFold modeling, HHpred sequence searches and DALI structural searches were used for the analysis of the major capsid protein, proteins supposedly participating in the adsorption stage, and endolysins (Figure 8, Supplementary Figures S2–S6). Structural modelling suggested that the major capsid protein (MCP) of phage NS-ph1 adopts a canonical HK97-like fold typical of tailed phage capsids. Structural superposition of the NS-ph1 MCP model with the experimentally determined MCP of the related *Pahexavirus* PA6 (PDB code 3JB5 chain A https://www.rcsb.org/structure/3JB5, accessed 2 December 2025) gave RMSD 0.612 Å, indicating a highly similar overall architecture (Figure 8a–c). In addition, beta-rich putative tail fiber proteins have a distinct two-domain structure, with the N-terminal domain similar to receptor-binding proteins of *Lactococcus* phages and the C-terminal domain showing similarity to different sugar-binding proteins (HHpred probability > 97%). The structural architecture of the predicted NS-ph1 TFP trimer was consistent with the trimeric RBP of *Lactococcus* phage p2 (PDB code 1ZRU [68]) (Figure 8d,e), including a comparable stacked arrangement of beta-rich domains. In this context, the gp17 model is consistent with a role as a host-interaction protein positioned on the virion periphery, where beta-rich domains often contribute to rigid ligand-binding surfaces [69,70,71]. The TFP of phage NS-ph2 is very similar to the TFP of NS-ph1 (pairwise identity 94.9%). AlphaFold-based modelling of the putative adhesion proteins encoded by phages NS-ph1 and NS-ph2 revealed that both proteins share a very similar elongated architecture, despite only 18.8% pairwise amino-acid identity. If this protein forms a trimeric structure, an N-terminal alpha-helical region from each predicted monomer could form an extended coiled-coil-like stalk, followed by a C-terminal beta-rich globular domain (Figure 8f). This domain organization is also consistent with that observed in phage receptor-binding and adhesion proteins of phages infecting Gram-positive bacteria [72]. Finally, endolysins of NS-ph1 and NS-ph2 (Figure 8g,h) displayed broadly similar modular architectures, each comprising a compact globular domain connected to an extended, predominantly alpha-helical region. Notably, the NS-ph1 endolysin model includes an additional N-terminal low-structure extension relative to NS-ph2, whereas the overall fold of the globular domain and the long helical segment remain comparable between the two proteins, being consistent with the sequence similarity of these proteins. HHpred searches found distinct similarities of NS-ph1 and NS-ph2 endolysins with different N-acetylmuramoyl-L-alanine amidases (HHpred probability > 99%).

## 4. Discussion

Although *C. acnes* is a commensal microorganism and an opportunistic pathogen, its association with a range of diseases (from acne to implant-associated infections) underscores the limitations of existing therapeutic regimens [11,28]. This provides a clear rationale for developing alternative agents capable of selectively and effectively eradicating *C. acnes.*

In this study, we demonstrated the potential of strictly lytic bacteriophages for the highly specific control of *C. acnes*. To this end, two such bacteriophages, NS-ph1 and NS-ph2, were directly isolated from the skin microbiota of acne patients, following standard protocols [73]. Lytic activity was observed for both phages against a panel of clinical *C. acnes* isolates, including antibiotic-resistant strains, which underscores their therapeutic potential as alternatives to conventional antibiotics. The strict species-level specificity of the NS-ph1 and NS-ph2 was further confirmed by their inactivity against other *Cutibacterium* species, consistent with previous reports on other phages targeting this species [73]. While the tested strains were not genetically characterized, these results suggest a narrow spectrum of activity, which is a key advantage for targeted therapy aimed at preserving the commensal microbiome.

The infection dynamics of NS-ph1 and NS-ph2 further underscore their therapeutic potential. Adsorption assays demonstrated rapid initial binding to *C. acnes* cells, followed by a saturation phase typical of efficient virulent phages. One-step growth analyses revealed distinct but complementary replication strategies: NS-ph1 exhibited a longer latent period (~4 h) combined with a high burst size (70 virions per infected cell), whereas NS-ph2 displayed a shorter latent period (~2 h) with a more modest burst size (59 virions per cell). Notably, although the latent periods of NS-ph1 and NS-ph2 are consistent with the broader kinetic range previously reported for *C. acnes* phages [74,75], the burst size of NS-ph1 substantially exceeds values described for earlier isolates. Such differences suggest that NS-ph1 may be particularly effective in achieving robust phage amplification and sustained bacterial clearance, while NS-ph2 could contribute to a faster initial reduction in bacterial load. In combination, these phages may provide a functionally synergistic phage cocktail, capable of both rapid onset and durable suppression of *C. acnes* populations.

Comparative genomics and structure-based analyses provide mutually consistent support for placing NS-ph1 and NS-ph2 within the genus *Pahexavirus*. Intergenomic similarity placed both phages within the *Pahexavirus* cluster, with NS-ph1 showing 85.11–91.04% similarity to pahexaviruses and NS-ph2 showing 86.09–91.28% (Figure 5). The closest genomes were *Cutibacterium* phage vB_CacS_YGG for NS-ph1 and *Cutibacterium* phage PHL113M01 (ICTV-classified as *Pahexavirus PHL113M01*) for NS-ph2, while similarity to phage PA6 was 88.93% and 88.61%, respectively. Phylogenetic analyses based on the major capsid protein and the ATPase domain of the large terminase subunit further supported this placement, recovering NS-ph1 and NS-ph2 within the *Pahexavirus* clade and indicating the existence of a possible new taxon at the genus level or higher, related to pahexaviruses and represented by the currently unclassified *Cutibacterium* phage CA1NRNZ. Taken together with the ICTV-recommended demarcation thresholds for bacterial viruses (95% for species and 70% for genus), the obtained results support the assignment of NS-ph1 and NS-ph2 to two novel species within the genus *Pahexavirus*. Furthermore, both phages possess compact dsDNA genomes of similar size (about 29.2–29.5 kb), lack tRNA genes, and display a canonical modular organization typical of tailed dsDNA phages, including conserved packaging and morphogenesis regions and more variable loci associated with adsorption. The content and organization of the structural module appear streamlined, consistent with the small genome size. The tail module is likely close to a minimal siphovirus composition. At the same time, the replication module appears relatively elaborate but lacks an identifiable DNA polymerase gene, suggesting that pahexaviruses may rely on the host polymerase, thereby saving genome space.

Within the largely conserved virion morphogenesis module, the predicted tail fiber and adhesion proteins appear to be less conserved than core structural proteins across the analyzed *Pahexavirus* genomes, which is consistent with their expected role in adsorption and host-range determination [76]. In our phages, the AlphaFold-predicted structural architecture of the putative adhesion proteins is highly similar between NS-ph1 and NS-ph2 despite very low sequence identity (18.8%), supporting fold-level conservation of these proteins. Interestingly, the predicted fold of TFP is compatible with known RBP-like designs described for Gram-positive-infecting siphoviruses [68]. Together, these observations support the interpretation that the identified tail fiber/adhesion proteins are the most likely candidates for receptor recognition in NS-ph1 and NS-ph2, although experimental validation will be required. Both genomes encode predicted endolysins, and HHpred hits to N-acetylmuramoyl-L-alanine amidase-like proteins are consistent with a peptidoglycan hydrolase involved in the terminal lysis step of the phage cycle [77,78]. Amidase-class endolysins cleave the amide bond between N-acetylmuramic acid and L-alanine residues in peptidoglycan, which is a common catalytic route for phage lysis proteins [79]. In the AlphaFold models, the C-terminal region forms an extended helical segment, which can be implicated in cell-wall association.

The molecular genetic characteristics of phages NS-ph1 and NS-ph2 place them within the genus *Pahexavirus* (class *Caudoviricetes)*. From a biosafety perspective, a key requirement for phage therapy is the absence of genes associated with lysogeny, virulence, or antibiotic resistance [80]. The absence of such genes in NS-ph1 and NS-ph2, together with a strictly lytic life cycle, identifies these phages as safe candidates for the development of phage-based therapeutics, particularly for topical use in skin infections.

The present study is limited to in vitro evidence of efficacy. While these data represent an important and encouraging first step, they do not fully predict therapeutic performance in vivo. Therefore, a crucial direction for future research is to evaluate these agents in relevant animal models (e.g., models of skin abscesses or implant-associated infections caused by *C. acnes),* as demonstrated in prior work [40,42]. Such studies would allow assessment not only of antimicrobial efficacy in vivo but also of pharmacokinetic parameters and potential toxicity.

The stability assessment of bacteriophages NS-ph1 and NS-ph2 under physiologically relevant skin pH ranges (approximately 4.1–5.8) and across a range of temperatures demonstrated that both phages can maintain lytic activity for an extended period (up to three months). These findings distinguish the present work from previous studies, which have primarily focused on short-term resistance to extreme conditions [43,74,75]. Both phages demonstrated high stability across −80 ± 2 °C to 25 ± 2 °C; however, NS-ph1 exhibited greater stability at elevated temperatures (37 ± 2 °C and 45 ± 2 °C). Optimal retention of activity was observed in the neutral and slightly acidic pH range (5.5–7.5). These results confirm that the studied bacteriophages, particularly NS-ph1, possess satisfactory physicochemical properties for the development of topical formulations, while the identified differences highlight the need for individualized formulation strategies.

It is recommended that future research examine the stability of phages formulated in specific dosage forms (such as gels or creams) under conditions closely resembling real storage environments.

## 5. Conclusions

NS-ph1 and NS-ph2 are strictly lytic *Cutibacterium* acnes phages that lyse all tested clinical *C. acnes* isolates, adsorb rapidly and show distinct replication kinetics, retain infectivity during extended storage at room temperature, and possess compact dsDNA genomes lacking identifiable lysogeny-associated genes, toxins, or antibiotic resistance determinants. Comparative genomics places both phages within the genus *Pahexavirus* but below the species-level similarity threshold relative to currently described members, supporting their classification as two new species. Collectively, these properties expand the available *C. acnes* phage repertoire and justify downstream work on formulation, antibiofilm performance, and in vivo efficacy and safety.

## Figures and Tables

**Table 1:** Data on antibiotic sensitivity and phage destruction (EOP) of clinical isolates of *C. acnes*.

*C. acnes* Strain	Source	Date Collected	Phage Killing (EOP)		Antibiotic Sensitivity									
			NS-ph1	NS-ph2	Azithromycin	Ampicillin	Gentamicin	Doxyciclin	Claritromicin	Clindamycin	Levofloxacin	Tetraciclin	Erythromicin	Ciprofloxacin
1.1	Clinical isolates from acne patients’ biomaterials	5 July 2024			S	S	S	S	S	S	S	S	S	S
2.1		5 July 2024		HOST	R	S	R	S	R	R	S	S	R	S
6.1		5 July 2024			S	S	S	S	S	S	S	S	S	S
9.1		5 July 2024			S	S	S	S	S	R	S	S	S	S
10.1		5 July 2024			S	S	S	S	S	S	S	S	S	S
12.1		5 July 2024	HOST		S	S	S	S	S	S	S	S	S	S
20.1		20 November 2024			S	S	S	S	S	S	S	S	S	S
21.1		20 November 2024			S	S	S	S	R	S	S	S	S	S
24.1		20 November 2024			S	S	S	S	S	S	S	S	S	S
25.1		20 November 2024			S	S	S	S	S	S	S	S	S	S
26.1		12 December 2024			S	S	S	S	S	S	S	S	S	S
A1.1		12 December 2024			R	S	S	S	S	R	S	S	S	S
28.1		12 December 2024			S	S	S	S	S	S	S	S	S	S
30.1		12 December 2024			S	S	S	S	S	S	S	S	S	S
31.1		12 December 2024			R	R	R	S	S	S	S	S	S	S
32.1		13 December 2024			S	S	S	S	S	R	S	S	R	S
35.1		13 December 2024			S	S	S	S	S	S	S	S	S	S
37.1		13 December 2024			S	S	S	S	S	S	S	S	S	S
39.1		13 December 2024			S	S	S	S	R	R	S	S	S	S
40.1		13 December 2024			S	S	S	S	S	S	S	S	S	S
41.1		13 December 2024			S	S	S	S	S	S	S	S	S	S
43.1		13 December 2024			R	S	S	S	S	S	S	S	S	S
VL1		4 Mar 2025			R	S	R	S	R	R	S	S	S	S
45.1		4 Mar 2025			R	S	S	S	S	S	S	S	S	S
46.1		4 Mar 2025			S	S	S	S	S	R	S	S	S	S
ATCC 6919	ATCC Collection				No study									
ATCC 11827					No study									
EOP ≥ 1.000			0.100 < EOP < 1.000		0.001 < EOP < 0.099			EOP < 0.001			No Lysis			

S-sensitive; R- resistant (highlighted in gray); EOP- Efficacy of Plating.

## Data Availability

Data are contained within the article and the Supplementary Materials.

## Supplementary Materials

The following supporting information can be downloaded at: https://www.mdpi.com/article/10.3390/v18020214/s1, Figure S1: Maximum-likelihood phylogenetic trees inferred from 100 representative amino acid sequences of the endonuclease domain of the terminase large subunit. *Pahexavirus* clade is colored light-pink. The tree is rooted to *Streptococcus* phage Javan34 and *Lactococcus* phage proPhi7. Bootstrap support values are shown next to the corresponding nodes; nodes with bootstrap support lower than 50% are collapsed into polytomies. The scale bar indicates the expected number of substitutions per site. Figure S2: AlphaFold 3 confidence output for the predicted structure of the major capsid protein of *Cutibacterium* phage NS-ph1. The AlphaFold 3 model is shown on the left and colored by per-residue confidence. The predicted aligned error (PAE) matrix is shown on the right, summarizing the uncertainty in the relative placement of residue pairs. The overall predicted TM-score (pTM) is 0.78. Figure S3: AlphaFold 3 confidence output for the predicted trimeric model of the putative tail fiber protein gp17 of *Cutibacterium* phage NS-ph1. The AlphaFold 3 model is shown on the left and coloured by per-residue confidence. The predicted aligned error (PAE) matrix is shown on the right. The AlphaFold 3 summary reports pTM = 0.47 and ipTM = 0.43. Figure S4: AlphaFold 3 confidence output for the predicted trimeric model of the putative adhesion protein gp19 of *Cutibacterium* phage NS-ph1. The AlphaFold 3 model is shown on the left and coloured by per-residue confidence. The predicted aligned error (PAE) matrix is shown on the right. The AlphaFold 3 summary reports pTM = 0.24 and ipTM = 0.23. Figure S5: AlphaFold 3 confidence output for the *Cutibacterium* phage NS-ph1 endolysin. The AlphaFold 3 model is shown on the left and coloured by per-residue confidence. The predicted aligned error (PAE) matrix is shown on the right. The overall predicted TM-score (pTM) is 0.61. Figure S6: AlphaFold 3 confidence output for the *Cutibacterium* phage NS-ph2 endolysin. The AlphaFold 3 model is shown on the left and coloured by per-residue confidence. The predicted aligned error (PAE) matrix is shown on the right. The overall predicted TM-score (pTM) is 0.66.
