# Supplementary figures and images for: Isolation and Genomic Characterization of Two Lytic Cutibacterium acnes Phages Defines Two Novel Pahexavirus Species
Source: Viruses. 2026 Feb 6;18(2):214. doi: 10.3390/v18020214 (PMC12945031; doi:10.3390/v18020214)

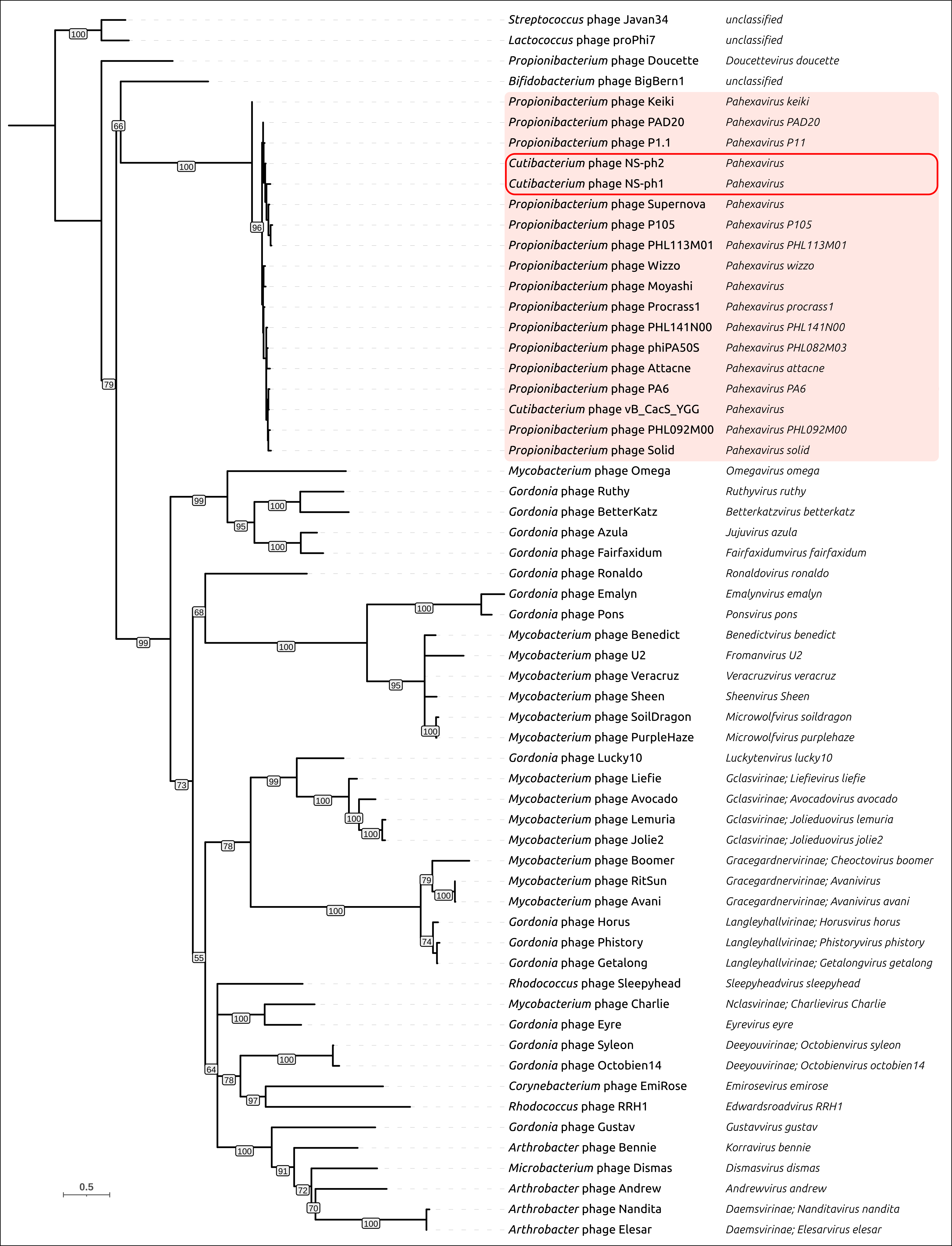

Supplement: Supplementary file 1 [file viruses-18-00214-s001.zip › Supplementary_Figure_S1.png]

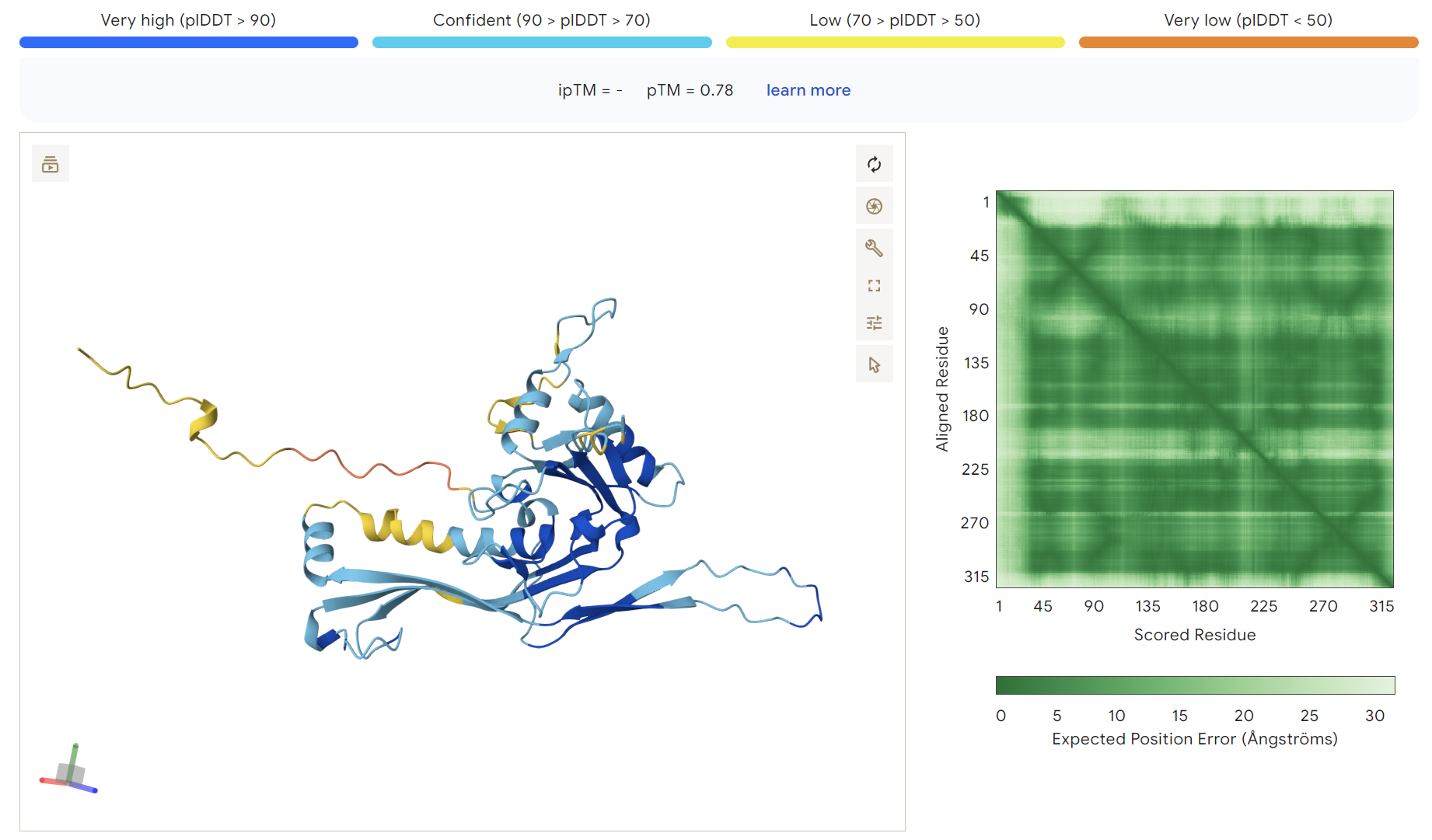

Supplement: Supplementary file 1 [file viruses-18-00214-s001.zip › Supplementary_Figure_S2.png]

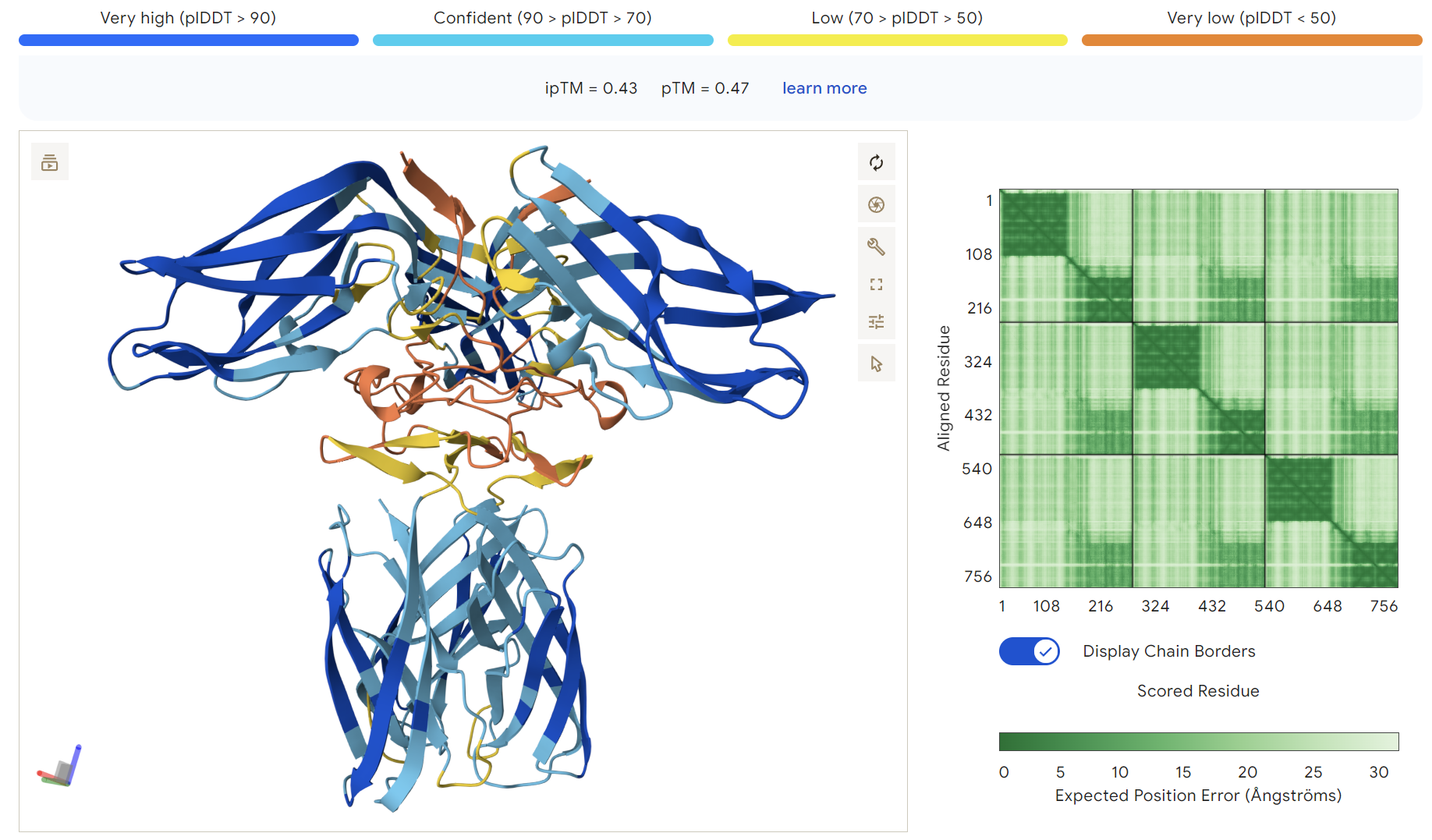

Supplement: Supplementary file 1 [file viruses-18-00214-s001.zip › Supplementary_Figure_S3.png]

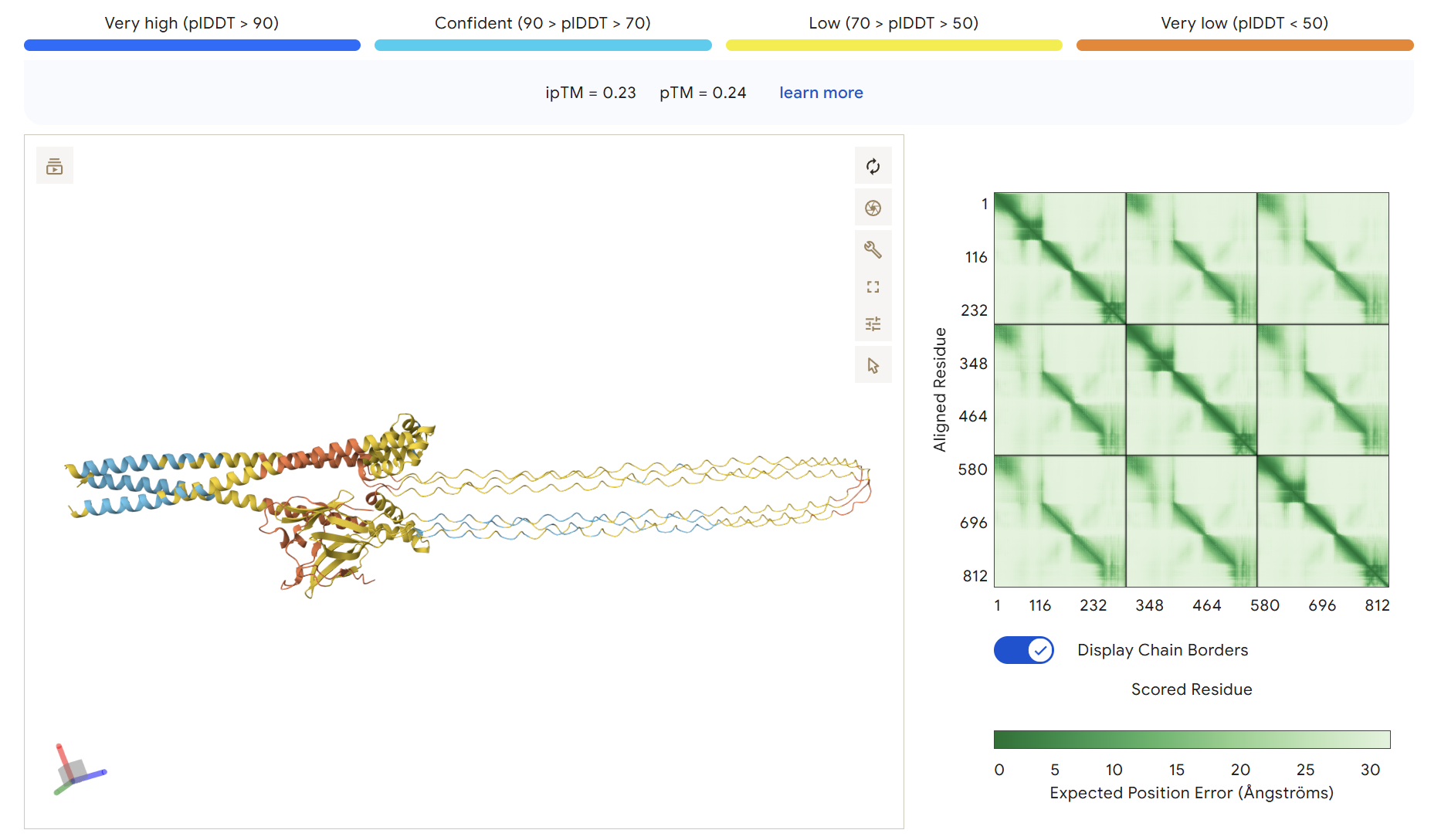

Supplement: Supplementary file 1 [file viruses-18-00214-s001.zip › Supplementary_Figure_S4.png]

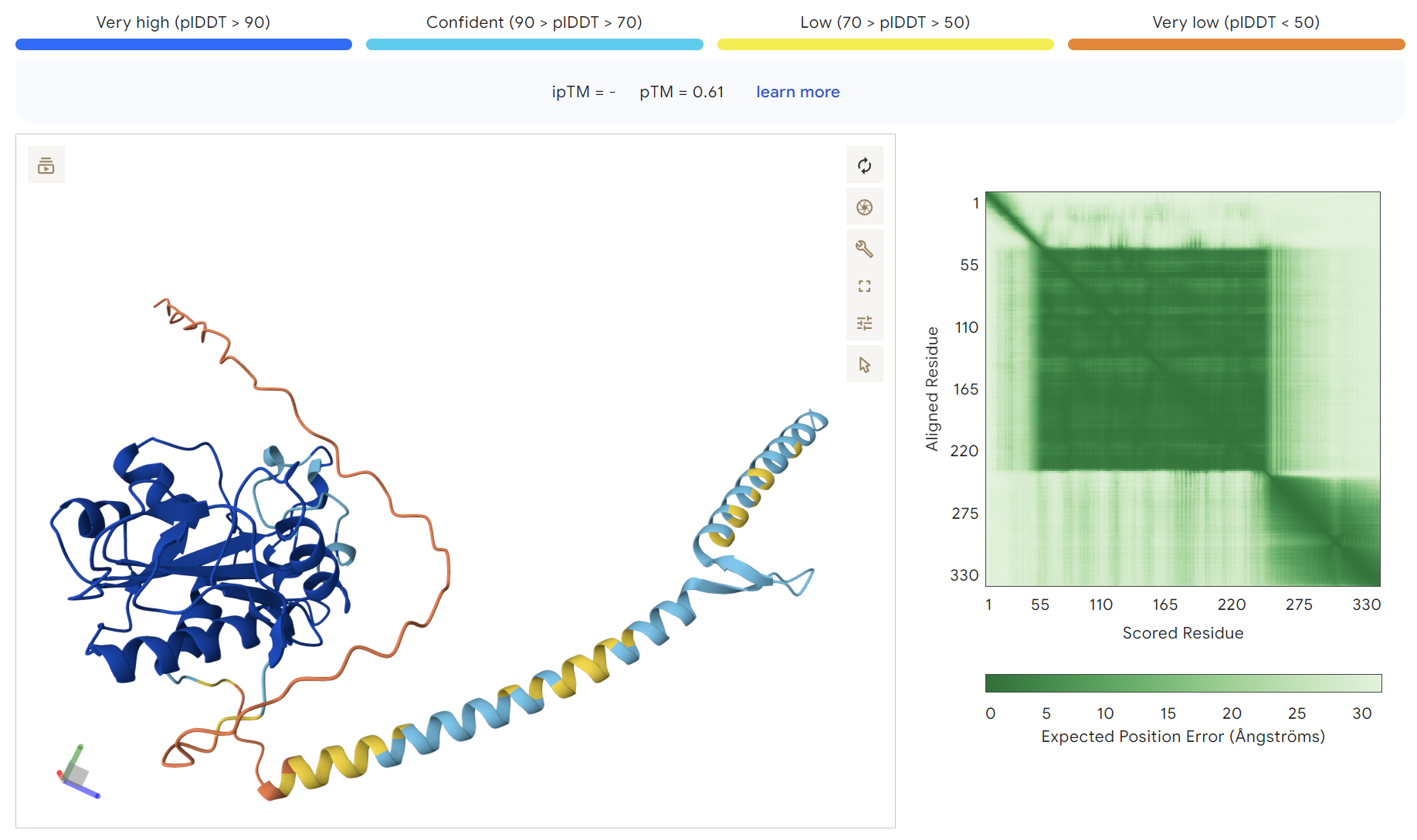

Supplement: Supplementary file 1 [file viruses-18-00214-s001.zip › Supplementary_Figure_S5.png]

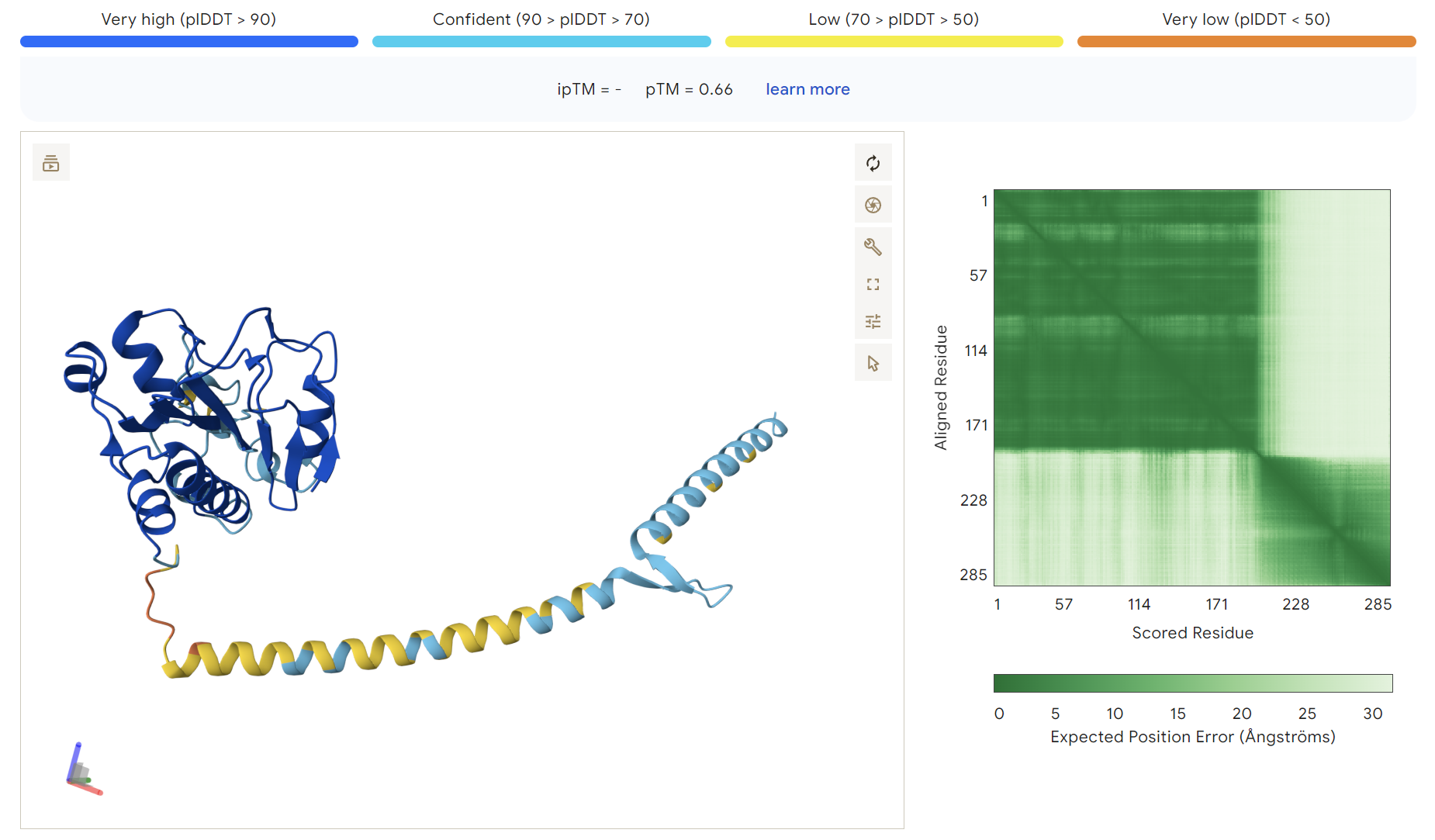

Supplement: Supplementary file 1 [file viruses-18-00214-s001.zip › Supplementary_Figure_S6.png]
